# Protective effects of indomethacin and dexamethasone in a goat model with intrauterine balloon aortic valvuloplasty

**DOI:** 10.1186/1423-0127-19-74

**Published:** 2012-08-13

**Authors:** Kaiyu Zhou, Gang Wu, Yifei Li, Liang Zhao, Rong Zhou, Qi Zhu, Xupei Huang, Dezhi Mu, Yimin Hua

**Affiliations:** 1 Department of Pediatric Cardiology, Second University Hospital and West China Medical School, Sichuan University, Chengdu, 610041, China; 2 Department of Obstetrics and Gynecology, Second University Hospital and West China Medical School, Sichuan University, Chengdu, 610041, China; 3 Department of Ultrasound Cardiography, Second University Hospital and West China Medical School, Sichuan University, Chengdu, 610041, China; 4 Department of Pediatrics, Second University Hospital and West China Medical School, Sichuan University, Chengdu, 610041, China; 5 Key Laboratory of Obstetric & Gynecologic and Pediatric Diseases and Birth Defects of Ministry of Education, Second University Hospital and West China Medical School, Sichuan University, Chengdu, 610041, China; 6 Program for Yangtze River Scholars and Innovative Research Team in University at West China Second University Hospital and West China Medical School, Sichuan University, Chengdu, 610041, China; 7 Department of Biomedical Science, College of Medicine, Florida Atlantic University, Boca Raton, FL, 33431, USA; 8 Department of Pediatric Cardiology, West China Second University Hospital, Sichuan University, No. 20, 3rd section, South Renmin Road, Chengdu, 610041, China

**Keywords:** Intrauterine balloon aortic valvuloplasty, Fetal goat, Gestational outcome, Ultrastructure, Hemodynamic, Indomethacin, Dexamethasone

## Abstract

**Background:**

Intrauterine balloon aortic valvuloplasty (IUBAV) has been used for critical aortic stenosis. However, it is necessary to determine the fetal impairments such as preterm birth after this approach and to find a way to prevent or reduce them.

**Methods:**

In the present study, we evaluated the therapeutic value of indomethacin (IDM) and dexamethasone (DXS) on reducing the preterm birth rate in experimental goats after IUBAV.

**Results:**

Our results indicated that the administration of IDM/DXS significantly reduced the rate of premature birth. IDM/DXS treatment led to preservation of myocardial ultrastructure with less damage, and amelioration of the fetal and placental circulation. Furthermore, we found that norepinephrine (NE) level was positively associated with the degree of myocardial damage. IDM/DXS administration led to a significant decrease of operation-induced increase of NE levels, which may be associated with the protective effects of IDM/DXS. Lastly, we found that the administration of IDM/DXS did not induce the risk of ductus arteriosus closure or slow down fetal growth.

**Conclusions:**

Our results indicate that IDM/DXS promotes a better gestational outcome at least partially by reducing stress response during and after the operation of IUBAV in the goat model. IDM/DXS may be a useful application in human patients during IUBAV intervention.

## Background

Most severe congenital heart defects have a poor prognosis if intervention is not received. Prenatal diagnosis and intervention of certain defects may correct the primary lesion and subsequently prevent the secondary lesions [1-6]. Several studies have demonstrated that the surgical techniques can ensure the mother’s safety in most cases [7-12]. Recently, ultrasound-guided intervention has been introduced to the surgery of intrauterine cardiac intervention (IUCI), which allows the intervention to be achieved without opening of uterus and establishing extracorporeal circulation [7-14].

Since the first report of intrauterine balloon aortic valvuloplasty (IUBAV) in 1991 [10], several groups have published their studies of prenatal cardiac interventions for aortic valve stenosis [7-13]. In these cases, the access to the fetal heart has been achieved by a direct ultrasound-guided strategy. However, even with successful intervention, fetal impairments induced by the novel approach are still unclear [7,13]. Recently, we established an experimental goat model of IUBAV and evaluated the operation-induced fetal metabolic and hemodynamic change, stress response, histological change in critical organs, as well as mother’s safety and gestation outcomes in the fetal and mother goats [15]. We found that the IUBAV operation had significant and immediate impact on the fetus and induced a strong stress response, which might not cause pathological changes in critical organs, but might disrupt the continuation of normal gestation in the long term.

We hypothesized that successful control of uterine contraction and suppress of stress response may enhance the post-procedure fetal states. Since indomethacin (IDM) can reduce uterine contraction and has been used to prevent preterm birth [16,17] and dexamethasone (DXS) is used to inhibit stress responses and also increases microvillous membrane formation of the newly generated syncytial layer, which may increase fetal growth, prevent preterm labor, and help the continuation of affected pregnancy [18,19], in the present study, we tested our hypothesis by evaluating the therapeutic value and potential risk of IDM and DXS in a goat model with IUBAV.

## Methods

### Animal and experimental group

Thirty-two pregnant goats with twin gestation in 2nd- and 3rd- trimester [112–123 days (term: 150 days), weight, 36.5–44.0 kg] and fifteen normal female goats without pregnancy (weight, 29.5–41.0 kg) were purchased from Daye Livestock & Poultry Farms (Ziyang, Sichuan). This study was completed in accordance with the guidelines on the care and use of animals for research purposes by the Animal Care and Use Committee of Sichuan University.

Pregnant goats were used to study the therapeutic value of IDM and DXS to control preterm delivery and its risk (Preterm birth was defined as birth before 135 gestational days). The gestational age and body weight of fetuses were estimated on the basis of pregnant date and further confirmed by measuring biparietal diameter (BPD) and femur length (FL). According to the treatment, these goats were divided into control and treatment groups (A and B groups, n = 16/group). The control group received placebos and treatment group received indomethacin (IDM, orally, 1.5-2 mg/kg/day) starting at 7 days before operation, and dexamethasone (DXS, intracardial injection into left ventricle, 1 mg/kg) during the IUBAV process. The fetus on the right side of mother abdomen was selected to receive IUBAV, and the one on left side served as control. Accordingly, fetuses were respectively defined as IUBAV and control groups (n = 16/group).

According to the combination of treatment and operation, the fetal goats were named as “no treatment control without IUBAV (A-c)”, “no treatment but with IUBAV operation (A-o)”, “treatment control without IUBAV operation (B-c)” and “treatment with IUBAV operation (B-o)”. The baseline of the enrolled fetuses was similar in the four groups, which included gestational day [d, mean ± SEM, 109.33 ± 1.99(A-c), 112.35 ± 2.61 (A-o), 107.66 ± 2.36(B-c), and 110.94 ± 2.94(B-o) respectively, p =0.196], biparietal diameter (mm, mean ± SEM, 42.36 ± 1.08, 45.51 ± 1.19, 44.21 ± 1.41, and 44.79 ± 0.99 respectively, p = 0.071), and aorta diameter (mm, mean ± SEM, 6.10 ± 0.13, 6.35 ± 0.16, 6.15 ± 0.15, 6.25 ± 0.15 respectively, p = 0.357). Eight pregnant goats (4 from A and 4 from B group) underwent Cesarean section (C-section) at 3 hour after the operation. Another eight pregnant goats (4 from A and 4 from B group) underwent Cesarean section (C-section) 3 days after operation. Hearts from those fetuses were collected. Two samples from each heart were taken from left ventricular front wall and muscular septum. Tissues were fixed with glutaraldehyde followed by osmium tetroxide. After being washed with PBS, the tissues were dehydrated in alcohol series, embedded in epoxy resin, sectioned at a thickness of 70 nm, stained with uranyl acetate and lead citrate. The resultant sections were subjected to examination for ultrastructure using a transmission electron microscope (H-7650, Hitachi, Japan).

Mitochondrial damage was assessed using FLAMENG grading system [20]: 0 = normal matrix granules without visible damage; 1 = loss of matrix granules and light clearing of matrix; 2 = moderate clearing of matrix, moderate swelling, and partial fragmentation of cristae; 3 = severe clearing, severe swelling, loss of cristae; and 4 = amorphous dense granules. Mitochondrial damage was examined at 5 microscopic fields for each tissue section. The average of the mitochondrial damage grading was used as damage index.

In the study on the effect of exogenous norepinephrine (NE) on myocardial ultrastructure in normal goats, saline or NE was intravenously infused to five groups of anesthetized goats (n = 3) at a dose of 1, 5, 10 and 30 μg/ kg/min for one hour (which was based on one-hour IUBAV operation), respectively, and accordingly named as control group (saline), NE1, NE5, NE10 and NE30. The blood samples were collected before the infusion, at 15, 30, 45, and 60 minutes following the initiation of infusion, as well as 30, 60, 120, and 180 minutes after the completion of infusion. Hearts were collected from the control, NE1, NE5 and NE10 groups 3 hours after the completion of NE infusion. All goats in NE30 group showed serious ventricular arrhythmias and were sacrificed when life-ending signs were observed. Hearts of goat of NE30 group were collected about 30 min after completion of NE infusion.

### Surgery

The goats were prepared as previously reported [15]. Briefly, pregnant goats were fasted for 36 hours, forbidden from drinking for 12 hours and anesthetized using Atropine (i.m., 0.03–0.05 mg/kg), Ketamine (i.m., 10 mg/kg), diazepam (i.v.p., 0.5–1 mg/kg) and infusion of 2.5% chloralhydrate (35–50gtt/min). Ketamine was added (3 mg/kg) every one hour. Diazepam (i.v.p., 0.3–0.5 mg/kg) was given when necessary. The fetus was sedated and anesthetized through the placental circulation plus intramuscular injection of Ketamine (10 mg/kg). During the entire surgery, mother goats were maintained with a spontaneous respiration and were supported with 40–60% oxygen and Ringer solution. Fetuses and mothers were monitored under ultrasound and electrocardiogram, respectively.

For the IUBAV operation, pregnant goats were placed in the supine position, following skin preparation and the abdominal wall was opened to expose part of uterus under aseptic conditions. With the continuous guidance of ultrasound (Mindray M5 color Diagnostic Ultrasound System, China), an appropriate puncture site and ultrasonic plane was selected. A trocar with 4 F hemostatic sheath and 18 gauge puncture needle (PTC-B, Hakko Co., Ltd., Japan) was introduced through the uterine wall, fetal chest wall, and into the fetal left ventricle (LV). The puncture needle was placed in the direction of blood flow. After the core needle was pulled out, a coronary guide wire (0.014 in, Abbott, Ltd., USA) was inserted until it reached the descending aorta. Following the guide wire, hemostatic core and sheath were inserted into the LV. Finally, the hemostatic core was pulled out and replaced with a balloon catheter (5–7.0 mm × 15 mm, Tyshak II NuMED, Inc., USA). The balloon was dilated 2 times at the level of the aortic annulus. The blocking time of blood flow in each balloon expansion was approximately 2 seconds. The control fetuses did not undergo the puncture and cardiac intervention. The total time from anesthesia to the close of the abdominal opening was about 1.5-2 hours. Lincomycin hydrochloride was intravenously infused (20 mg/kg) to mother goats intraoperatively and also used for intraperitoneal (1.2 g/goat) rinse at the end of the surgery plus postoperative administration (10 mg/kg/day for 7 days). Left ventricular systolic pressure (LVSP) was measured through puncture needle or trocar at different time points, with an extension tube connected to pressure monitor (Mindray PM-7000 portable multi parameter monitor, China).

### Sample collection and data analysis

Based on our previous study, the following five time points were chosen in the current study: before IUBAV (the time after the needle successfully was inserted into the heart but before the dilation of balloon, named as “Pre”), after IUBAV (after the operation was completed, named as “Post”), 3 hours and 3 days post-operation (namely, 3 h-post and 3d-post, respectively), and 24 hours after birth (named as Neo). The data for heart rate, ratio of peak blood flow between systolic and diastolic phase (S/D) of aorta and umbilical artery, diameter and blood flow peak velocity in ductus arteriosus were obtained from echocardiographic results. Left ventricular systolic pressure (LVSP) for operated fetus was measured through balloon catheter during IUBAV operation. For control goats, cardiac puncture with PRC-B 18-20 G (Hakko Co., Ltd., Japan) was performed, and LVSP was recorded via a biomedical recording system (PM-8000 Mindray, Shenzhen, China). Serum from fetus or normal adult goat were collected at designated time points and subjected to measurement of NE concentration using routine clinical sample analysis method and procedure.

### Statistical analysis

The SPSS program software (version 13.0, SPSS Inc., USA) was used for statistical analysis. Quantitative data were expressed as Mean ± SEM. One way ANOVA was used to analyze the difference among groups and Fisher Exact Test was used for two group comparisons. The differences were considered significant when *p <*0.05.

## Results

### IDM/DXS treatment reduced the rate of preterm delivery following IUBAV operation

**Figure 1:**
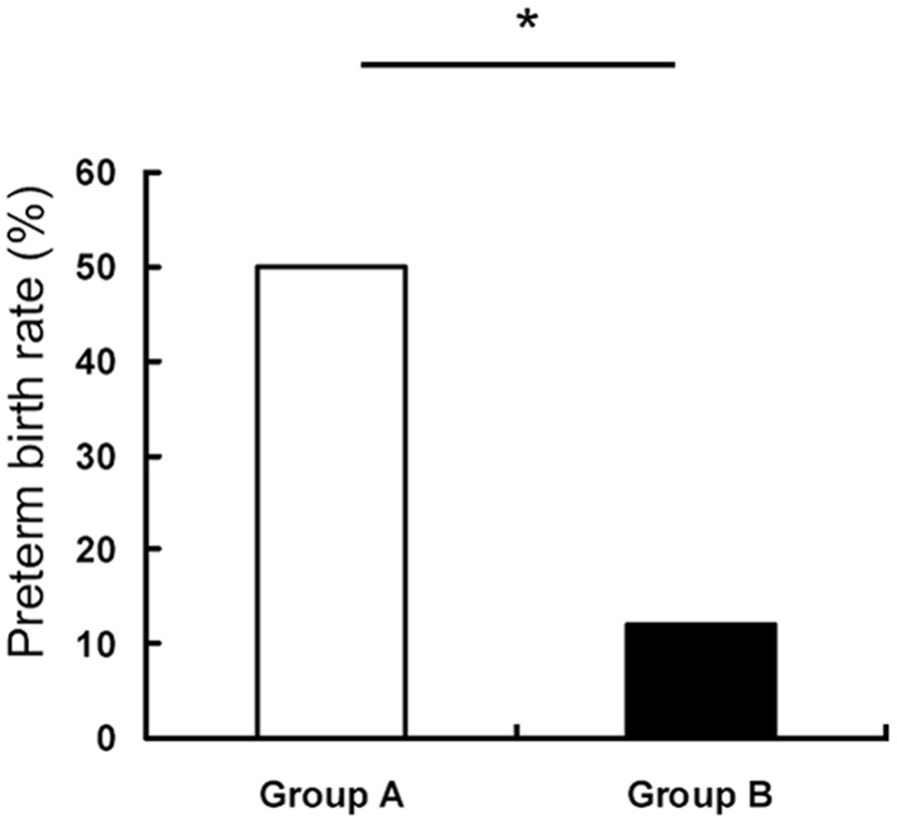
**Administration of IDM/DXS decreased the rate of preterm delivery following IUBAV operation.** Two groups of pregnant goats received IUBAV were daily monitored up to the time of natural delivery. The preterm birth rate was calculated in control (group A without IDM/DXS treatment, empty bar) and IDM/DXS treated groups (group B, black bar).

To determine whether IDM/DXS is beneficial for the improvement of the gestation outcome following IUBAV, we monitored both treatment and control groups of pregnant goats up to the time of natural delivery. In contrast to 50% (4 of 8) preterm delivery in the pregnant goats that did not receive IDM/DXS treatment, only 12.5% (1 out 8) preterm delivery was observed in the pregnant goats received IDM/DXS treatment (Figure 1).

### IDM/DXS treatment ameliorated hemodynamic during and after IUBAV

**Figure 2:**
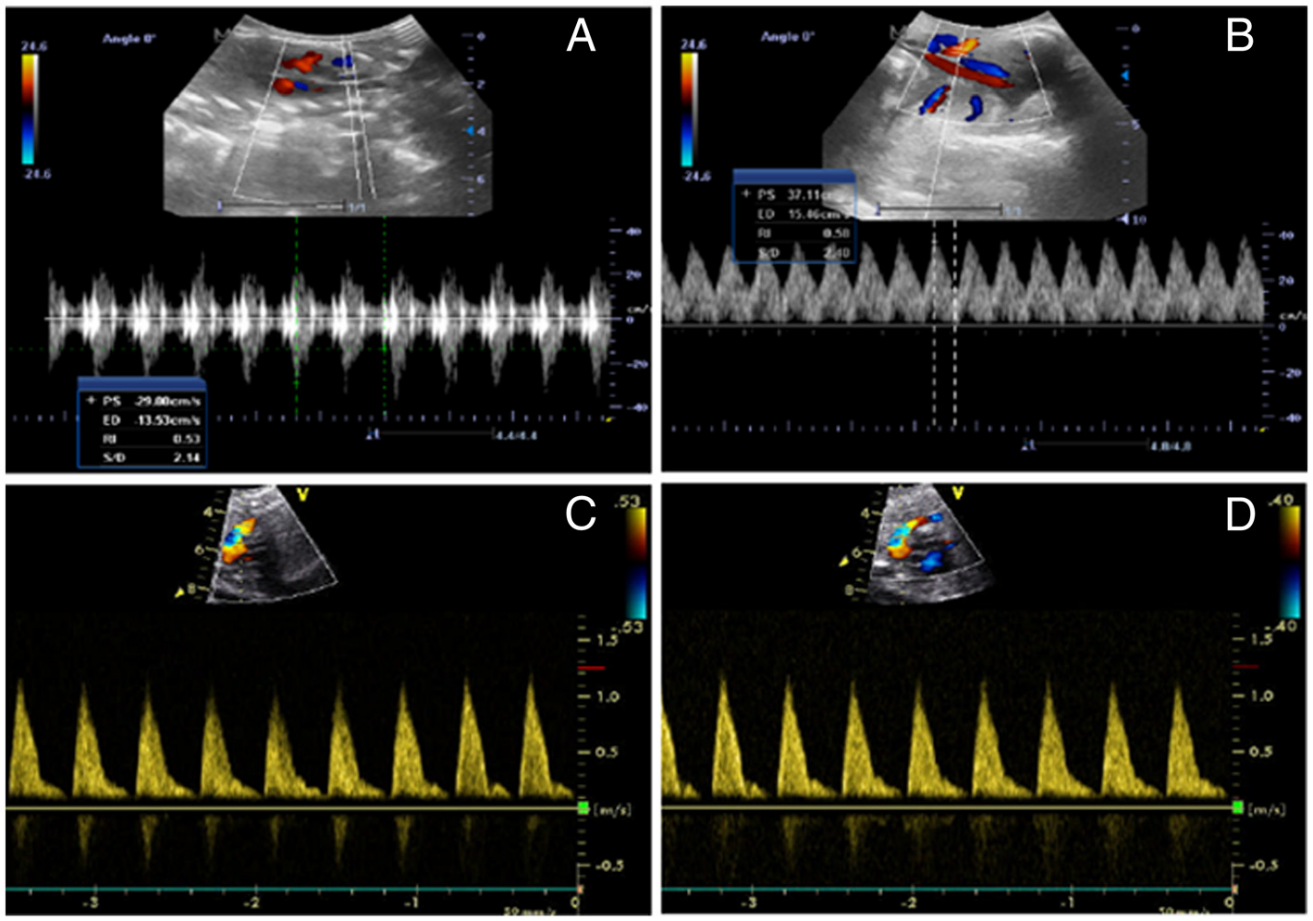
**Hemodynamic measurements on experimental animals with ultrasound.** Upper panel: representative echocardiographic results showing blood flow and S/D in fetal aorta (**A**) and umbilical artery (**B**). Lower panel: representative spectrum of blood flow in ductus arteriosus of fetus without (**C**) or with (**D**) IDM/DXS treatment.

To evaluate whether IDM/DXS treatment impacts the fetal hemodynamic response to the operation, we measured heart rate, left ventricular systolic pressure (LVSP), ratio of peak blood flow at systolic and diastolic phase (S/D) of aorta and umbilical artery, diameter and blood flow peak velocity in ductus arteriosus at designated time points (Figure 2 and Tables 1, 2, 3 and 4). As shown in Table 1, the operation induced a significant increase of heart rate compared to the preoperational levels in all fetuses either immediately (time point: Post) or 3 days post operation. IDM/DXS administration alone did not alter the HR (group B). LVSP was decreased at the time of IUBAV completion compared to the preoperational levels in both control and IDM/DXS groups (Table 2). The decreased LVSP returned to the baseline 3 hours after the operation. However, the LVSP in IDM/DXS group with IUBAV (B-o group) was higher than that in the group without IDM/DXS treatment but with IUBAV (A-o), and a quicker return of LVSP to baseline was revealed, which might be caused by IDM/DXS treatment. Aortal S/D in goat fetuses was significantly increased in both groups with or without IUBAV and it did not return to the preoperational level until 3 days after the operation (denoted by * in Table 3). Noticeably, the increase of aortal S/D in fetuses with IUBAV (group A-o and B-o) showed a greater extent than that in the counterpart twins without IUBAV (groups, A-c, and B-c), and this difference was detected at the time of IUBAV completion and 3 hour post operation (denoted by p1 and p2, respectively, in Table 3). Encouragingly, IDM/DXS administration appears to inhibit the aortal S/D increase in fetuses from both groups with or without IUBAV (denoted by p3, and p4, respectively, in Table 3), and this effect was also shown at the time of IUBAV completion and 3 hour post operation. Consistently, umbilical artery S/D of goat fetuses showed the same change pattern as aortal S/D following IUBAV and IDM/DXS treatment (Table 4), suggesting that IDM/DXS treatment significantly ameliorated both fetal and placental circulation..

**Table 1:** Heat rate of goat fetuses and neonates before and after operation (mean ± SEM)

**Group**	**Pre (n = 16)**	**Post (n = 16)**	**3 h-Post (n = 16)**	**3d-Post (n = 12)**	**Neo**^**▴**^
A-c	136 ± 5	197 ± 7*	188 ± 5*^#^	165 ± 6*^#§^	124 ± 10
A-o	144 ± 6	206 ± 6*	191 ± 9*^#^	168 ± 6*^#§^	119 ± 11
B-c	138 ± 4	188 ± 8*	175 ± 6*^#^	158 ± 8*^#§^	126 ± 11
B-o	135 ± 6	196 ± 7*	183 ± 7*^#^	161 ± 6*^#§^	127 ± 8
*p*	0.163	0.067	0.085	0.112	0.125

*significant difference compared to Pre, ^#^significant difference compared to Post, ^§^significant difference compared to 3 h-Post; ^▴^n = 4 in groups A-c and A-o; n = 7 in groups B-c and B-o.

**Table 2:** Left ventricular systolic pressure of goat fetus and neonates (mmHg) (mean ± SEM)

**Group**	**Pre**	**Post**	**3 h-Post**	**3d-Post**	**Neo**^**▴**^
	**(n = 16)**	**(n = 16)**	**(n = 4)**	**(n = 4)**	
A-c	--	--	27.36 ± 1.66	25.92 ± 1.61	56.17 ± 2.98
A-o	24.32 ± 0.63	19.61 ± 1.04*	22.34 ± 1.33^#^	26.11 ± 2.31^#^	58.92 ± 3.39
B-c	--	--	27.62 ± 2.12	27.73 ± 1.87	63.13 ± 2.19
B-o	26.24 ± 0.84	23.51 ± 0.49*^§^	25.37 ± 1.98^#§^	25.47 ± 1.43^#^	59.45 ± 1.87
*p*	0.072	**0.003**	**0.026**	0.087	0.127

*significant difference compared to Pre; ^#^significant difference compared to Post; ^§^significant difference compared to A-o; -- = not measured; ^▴^n = 4 in groups A-c and A-o; n = 7 in groups B-c and B-o. *p <*0.05 is given in bold.

**Table 3:** Aortic S/D of goat fetus and neonates (mean ± SEM)

**Group**	**Pre (n = 16)**	**Post (n = 16)**	**3 h-Post (n = 16)**	**3d-Post (n = 12)**	**Neo**^**▴**^
A-c	2.31 ± 0.08	2.87 ± 0.06*	2.59 ± 0.07*^#^	2.44 ± 0.04^#^	2.19 ± 0.12
A-o	2.45 ± 0.06	3.44 ± 0.08*	3.11 ± 0.06*^#^	2.37 ± 0.06^#§^	2.36 ± 0.12
*p1:*	0.165	**0.0001**	**0.0001**	0.344	0.035
B-c	2.39 ± 0.07	2.69 ± 0.06*	2.34 ± 0.03^#^	2.33 ± 0.06^#^	2.25 ± 0.06
B-o	2.49 ± 0.06	3.01 ± 0.09*	2.88 ± 0.05*^#^	2.53 ± 0.07^#§^	2.33 ± 0.07
*p2*	0.477	**0.007**	**0.0001**	0.021	0.42
*p3*	0.453	**0.031**	**0.002**	0.131	0.713
*p4*	0.631	**0.002**	**0.003**	0.069	0.829

*significant difference compared to Pre; ^#^significant difference compared to Post; ^§^significant difference compared to 3 h-Post; *p*1: A-o vs. A-c; *p*2: B-o vs. B-c; *p*3: B-c vs. A-c; *p*4: B-o vs. A-o. ^▴^n = 4 in groups A-c and A-o; n = 7 in groups B-c and B-o. *p <*0.05 is given in bold.

**Table 4:** Umbilical artery S/D of goat fetus and neonate (mean ± SEM)

**Group**	**Pre (n = 16)**	**Post (n = 16)**	**3 h-Post (n = 16)**	**3d-Post (n = 12)**	**Neo**^**▴**^
A-c	2.21 ± 0.03	2.44 ± 0.06*	2.59 ± 0.06*	2.46 ± 0.04*	1.94 ± 0.11
A-o	2.25 ± 0.05	3.11 ± 0.07*	2.87 ± 0.04*^#^	2.67 ± 0.04*^#§^	2.15 ± 0.08
*p1*	0.512	**0.0001**	**0.001**	**0.001**	0.07
B-c	2.09 ± 0.06	2.55 ± 0.05*	2.54 ± 0.05*	2.23 ± 0.06	2.11 ± 0.06
B-o	2.11 ± 0.05	2.83 ± 0.05*	2.65 ± 0.07*^#^	2.56 ± 0.06*^#^	2.08 ± 0.07
*p2*	0.746	**0.001**	0.541	**0.001**	0.727
*p3*	0.071	**0.034**	0.066	**0.002**	0.153
*p4*	0.063	**0.002**	**0.016**	0.936	0.516

*significant difference compared to Pre; ^#^significant difference in contrast to Post; ^§^significant difference compared to 3 h-Post; *p*1: A-o vs. A-c; *p*2: B-o vs. B-c; *p*3: B-c vs. A-c; *p*4: B-o vs. A-o. ^▴^n = 4 in groups A-c and A-o; n = 7 in groups B-c and B-o. *p <*0.05 is given in bold

### Myocardial ultrastructure change following IUBAV with or without IDM/DXS treatment

**Figure 3:**
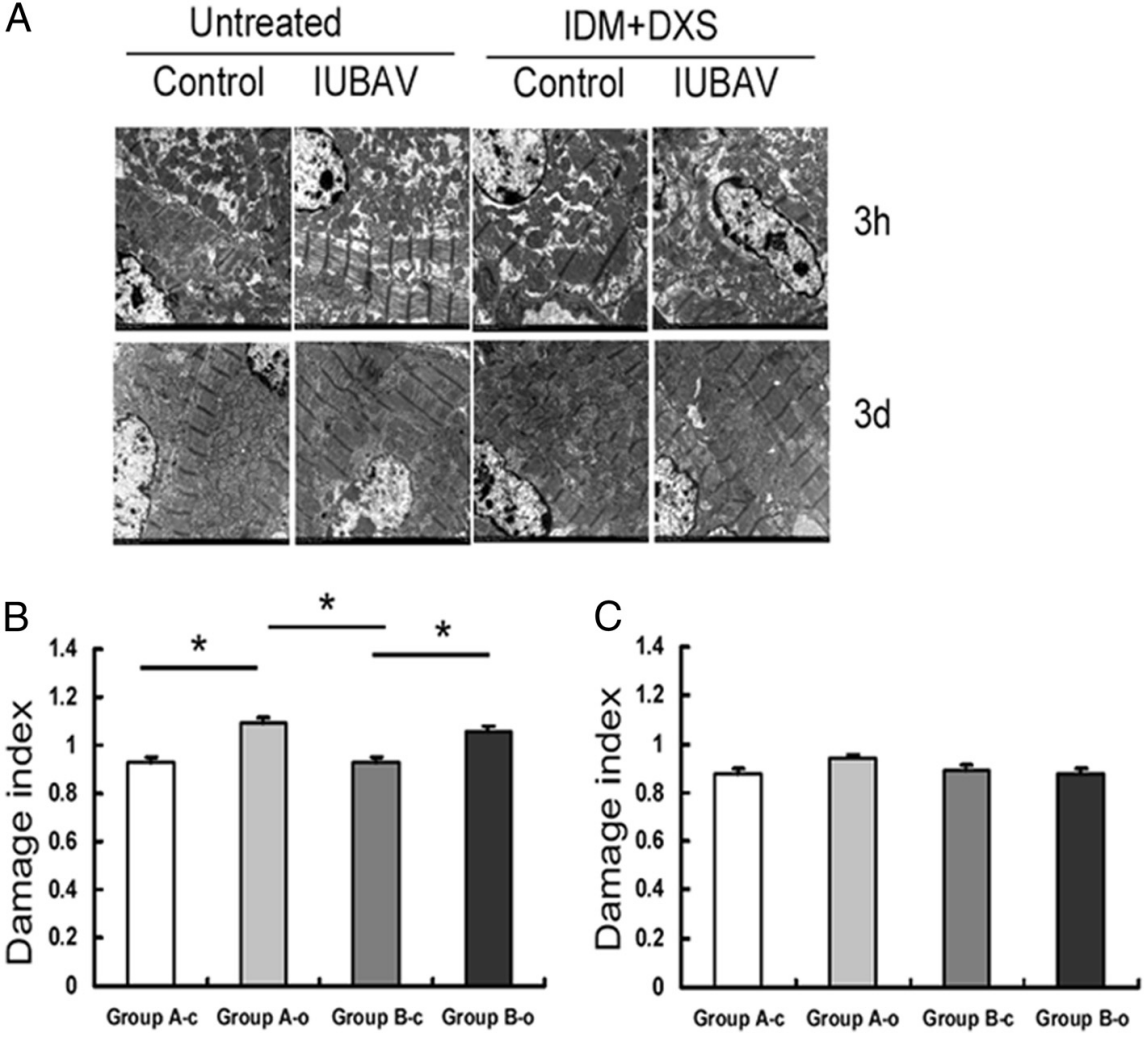
**Myocardial ultrastructure of goat fetuses without or with IDM/DXS treatment.A**: Representative micrographs of four groups of goat fetal myocardial tissues (×24000) at 3 hours (upper panel) and 3 days (lower panel) post operation. **B**: Quantitative results of mitochondrial damage at 3 hours post operation. **C**: Quantitative results of mitochondrial damage at 3 days post operation.

To further evaluate the impact of IUBAV on heart structure and function, we examined the ultra structure of the heart using an electron microscope in fetuses from four groups 3 hours and 3 days after the operation (Figure 3). The myocardial damages were analyzed based on the myocardial and mitochondria morphological changes [20]. As shown in Figure 3, myocardial vacuolization, degranulation of endoplasmic reticulum, and mitochondria damages were observed in the fetal hearts of four groups with a varied extent. Statistical comparisons revealed that significant differences existed between fetuses with IUBAV and fetuses without IUBAV (A-o vs. A-c, and B-o vs. B-c in Figure 3B) 3 hours after the operation. However, these differences were not detected on fetuses 3 days post-operation, suggesting that myocardial damage at ultrastructure level was reversible (Figure 3A low panel and 3C).

### IDM/DXS treatment reduced fetal NE level

Our previous study [15] revealed that fetal NE level was remarkably increased following IUBAV. Since NE has various effects on hemodynamics, we further determined whether IDM/DXS treatment could change the level and acting time of fetal serum NE after IUBAV operation. As shown in Table 5, while NE levels in fetuses with IUBAV was significantly higher than the preoperational level in both A-o and B-o groups at the time of IUBAV completion, 3 hours, and 3 days post operation, the magnitude of the NE increase was significantly less in IDM/DXS treatment groups (group B-o vs. A-o). And the duration of elevated NE was shorter in IDM/DXS groups than that in control groups. These results suggested that one of the beneficial effects of IDM/DXS was to control NE level and to reduce stress response and shorten NE acting time.

**Table 5:** Fetal serum NE concentration before, during and after IUBAV operation in the presence and absence of IDM/DXS treatment ( mean ± SEM )

**Group**	**Pre (n = 16)**	**Post (n = 16)**	**3 h-Post (n = 4)**	**3d-Post (n = 4)**
A-c	--	--	4515.91 ± 182.94	2251.22 ± 100.85 ^§^
A-o	1633.45 ± 61.87	9921.29 ± 223.68*	6436.81 ± 282.85 ^*#^	3133.58 ± 98.18 ^*#§^
B-c	--	--	3628.33 ± 194.56	2128.64 ± 132.35 ^§^
B-o	1542.08 ± 49.81	8246.54 ± 283.86*	4792.31 ± 389.67 ^*#^	2098.35 ± 134.48 ^*#§^
*p1*	n/a	n/a	**0.009**	**0.001**
*p2*	n/a	n/a	**0.001**	0.725
*p3*	n/a	n/a	**0.017**	0.487
*p4*	0.356	**0.001**	**0.015**	**0.001**

*significant difference compared to Pre; ^#^ significant difference compared to Post; ^§^significant difference compared to 3 h-Post; -- = not measured; n/a = not applicable; *p*1: A-o vs. A-c; *p*2: B-o vs. B-c; *p*3: B-c vs. A-c; *p*4: B-o vs. A-o. *p <*0.05 is given in bold.

### Myocardial ultrastructure change was associated with the level of NE

**Figure 4:**
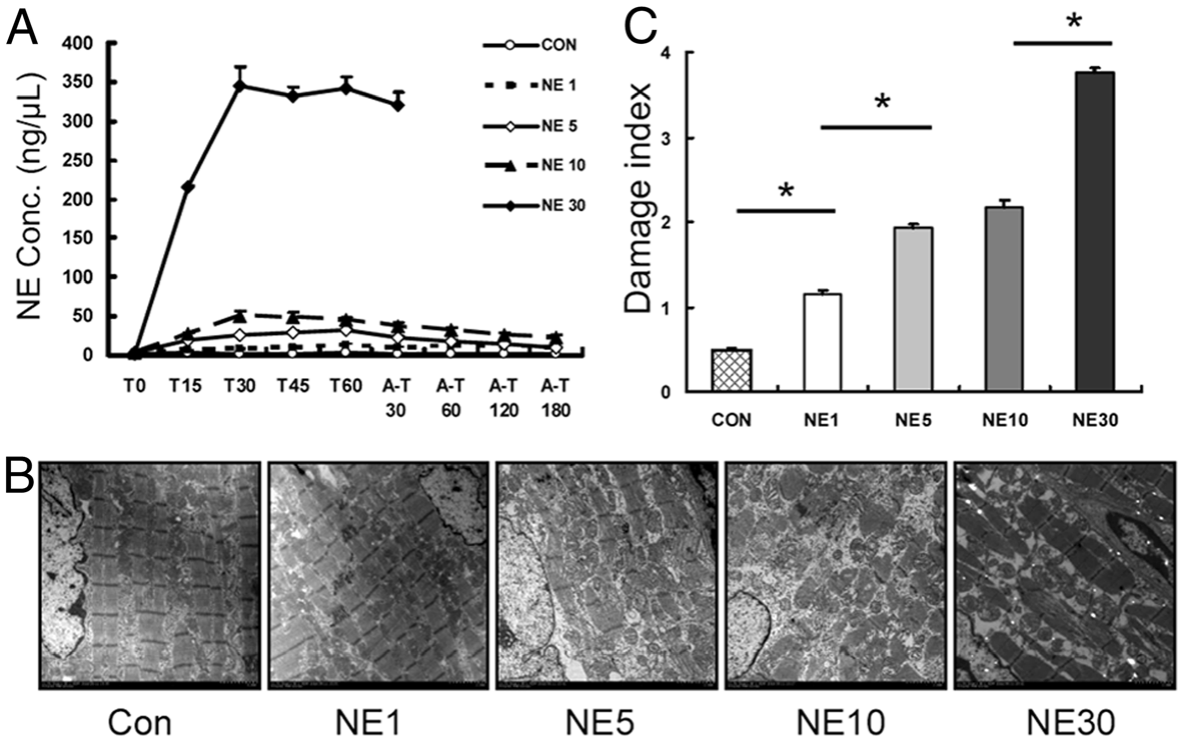
**The relationship between NE level and myocardial damage.A**: Starting from the initiation of intravenous infusion of saline or NE, goat blood samples were collected during (T0-T60) and after infusion (A-T30-180) at designated time points. Serum samples were subjected to measurement of NE level and each group was plotted in a curve (Con = saline, NE group was named according to the infused NE concentration. This group name is applicable for subfigure B and C). **B**: Representative micrographs of myocardial tissue of goats at 3 hours after the completion of NE for groups, Con, NE1, NE5 and NE10; at 30 min after NE infusion for group NE30 (×24000). **C**: Quantitative results of mitochondrial damage as observed in B.

It has been reported that high level of NE results in myocardial damage [21-23]. To assess the relationship between NE and myocardial damage in our goat model, we examined the myocardial ultrastructure and damage in five groups of the normal goats (n = 5) receiving infusion of NE at a variety of concentrations. As shown in Figure 4, exogenous infusion of NE led to an increase of serum NE level; and the artificial increase of NE level was correlated to the extent of the myocardial damage in a dose-dependent manner. In addition, fetal NE level and myocardial damage following IUBAV were comparable with the features of NE1 and NE5, especially closed to NE1, which were at low level and would result reversible damage *in vitro* and *in vivo* experiments.

### Evaluation of the safety of IDM/DXS administration in short- and long-terms

Since indomethacin has been used to promote closure of ductus arteriosus in pre-term newborns, which could be one side effect if used in the IUBAV [16,24], we further measured diameter and blood flow peak velocity in ductus arteriosus of goat fetuses to determine this effect of indomethacin. As shown in Table 6, the diameter and blood flow velocity of ductus arteriosus did not show significant difference among the fetuses from the four groups. Furthermore, we compared the body weight (BW) and hematocrit (Hct) among the full-term neonates from treated and untreated group with IDM/DXS, to determine the impact of IDM/DXS on development and growth. The parameters including Body weight [BW, kg, 2.36 ± 0.11 (A-c), 2.33 ± 0.13 (A-o), 2.34 ± 0.07 (B-c), 2.39 ± 0.12 (B-o) respectively, p = 0.806] and hematocrit (Hct, 0.38 ± 0.02, 0.39 ± 0.02, 0.41 ± 0.02, and 0.40 ± 0.02 respectively, p = 0.749) of full-term neonates (mean ± SEM), no significant differences were detected. These data suggest that IDM/DXS at administered dose in the current study can promote a better gestational outcome without introducing risk of ductus arteriosus closure or delaying fetal growth.

**Table 6:** Diameter (D) and blood flow peak velocity (V) in ductus arteriosus of goat fetuses (mean ± SEM)

**Group**	**Pre-treatment (n = 16)**		**Pre-procedure (n = 16)**		**3d-Post procedure (n = 12)**	
	**D(mm)**	**V(m/s)**	**D(mm)**	**V(m/s)**	**D(mm)**	**V(m/s)**
A-c	3.12 ± 0.11	1.06 ± 0.03	3.11 ± 0.06	1.14 ± 0.05	3.19 ± 0.11	1.12 ± 0.04
A-o	2.96 ± 0.11	1.13 ± 0.04	3.02 ± 0.09	1.21 ± 0.05	3.15 ± 0.10	1.17 ± 0.05
B-c	3.03 ± 0.08	1.09 ± 0.03	2.94 ± 0.10	1.15 ± 0.05	3.06 ± 0.09	1.24 ± 0.06
B-o	3.13 ± 0.11	1.07 ± 0.04	3.16 ± 0.09	1.18 ± 0.05	3.21 ± 0.09	1.09 ± 0.06
*p*	0.196	0.163	0.344	0.226	0.189	0.062

## Discussion

Intrauterine intervention for certain congenital heart defects, such as obstruction, may prevent the progression of the severity of the primary lesions and the development of secondary lesions [7-12]. Although the technical feasibility has been achieved in animal models as well as in clinical patients [13,25-28], the selection of the right patients in consideration of the long-term outcome of the fetus is still controversial. Our recent study [15] indicated that the IUBAV operation itself causes significant stress responses in fetuses of a goat model, which may in turn disrupt the normal course of gestation. Therefore, we extended our study to determine whether the combinatorial application of contraction inhibitor, indomethacin, and stress inhibitor, dexamethasone, could improve the gestational outcome. Furthermore, we tried to explore the protective effects of IDM/DXS on hemodynamics, myocardial ultrastructure, and stress response, which are not well addressed in human patients due to ethical concerns.

In the current study, we obtained several important findings. First, the administration of IDM/DXS significantly reduces the rate of premature birth. Second, IDM/DXS treatment leads to a preservation of myocardial ultrastructure with less damage and an amelioration of the fetal and placental circulation. Third, administration of IDM/DXS significantly decreases IUBAV operation-induced elevation of NE levels, which might be associated with myocardial ultrastructure changes, since the NE level is positively related with the degree of myocardial damages. Lastly, IDM/DXS at the dose that we used in our study can promote a better gestational outcome without introducing risk of ductus arteriosus closure or delaying fetal growth. Collectively, our data suggest that administration of IDM/DXS promotes a better gestational outcome by ameliorating fetal hemodynamic and reducing myocardial damages caused by IUBAV operation.

As a nonspecific inhibitor of cyclooxygenase (COX), indomethacin is capable of reducing uterine contraction by blocking the synthesis of prostaglandin (PG). It has been widely used as an agent to control premature birth [16,17]. In addition to its contraction-inhibiting effects, our study also shows that IDM is capable of reducing the operation-induced hemodynamic fluctuation in fetuses, which is consistent with other studies [29,30]. Dexamethasone is a synthetic corticosteroid widely used in serious or emergency situations, including surgery [18,19]. Because it enables the body to overcome stress by increasing the heart rate and contractility, leading to an increase of blood supply to essential organs, DXS was intracardially injected along with the application of indomethacin in the present study. We did not observe any side effects related to the administration of IDM/DXS under our study conditions. While we thought that the observed beneficial effects were derived from reduction of uterine contraction and decrease of stress responses, we can not exclude other mechanisms of the two agents that may be involved in inhibiting the production of other labor-initiating factors. For example, both IDM and DXS possess anti-inflammatory and immunosuppressive properties, which may contribute to the control of inflammatory responses to the tissue damage during operation and ultimately reduce the level of inflammatory mediators that may promote the labor initiation. In addition, IDM readily crosses the placenta and can reduce fetal urine production and the amount of amniotic fluid. DXS also increases microvillous membrane formation of the newly generated syncytial layer and thus facilitates the continuation of affected pregnancy [18,19]. In summary, the exact mechanisms underlying the beneficial effects of IDM/DXS treatment after IUBAV remain to be elucidated by further investigation.

It is well known that catecholamines have a variety of effects on cardiovascular system, depending on their concentration and acting time [31]. While it promotes myocardial hypertrophy and regulates vascular resistance at a low concentration, NE can cause myocardial apoptosis and necrosis at a high concentration both *in vitro* and *in vivo*[32,33]. Although fetuses during the IUBAV operation are anesthetized through the placental circulation, their stress responses are still significant and the “catecholamine storm” can directly result in the impairment of myocardial ultrastructure. To test this possibility, we conducted an experiment that was specifically designed to determine the relationship between serum NE levels and myocardial ultrastructure. Consistently, we observed a similar myocardial damage after infusion of exogenous NE. It strongly suggests that IUBAV-induced release of endogenous NE might be, at least partially, responsible for the myocardial damages in fetal hearts after IUBAV operation.

Through comparison, we found that fetal NE level and myocardial damage in IUBAV were comparable with that of infused exogenous NE at low level. The IUBAV-induced fetal myocardial damage seems to be transient and reversible without intervention. IDM/DXS treatment can significantly reduce the magnitude of the increased NE level, reduce the degree of myocardial damages and shorten the acting time of NE after IDM/DXS treatment. Although all the changes following IUBAV were combination effects by multiple factors, we only selected a representative index (NE) to research, because the direction and level of NE were well consistent with the stress reaction and other simulations of fetal lambs.

It is encouraging to observe the beneficial effects of IDM/DXS on the gestational outcome after IUBAV operation. However, there is still about 12.5% of preterm birth in our model, suggesting that IDM/DXS administration is still not sufficient to prevent all preterm birth following the IUBAV. Other potential causes of preterm birth remain to be identified and corrected. Asymptomatic infection may be a concern, which could be treated with antibiotics. In addition, we do not know if IUBAV could cause the fluctuation of progesterone level, which may be corrected by progesterone. Therefore, in addition to IDM/DXS, other tocolytics may also be useful to completely control the preterm delivery following the IUBAV. However, it should be cautious in evaluation of the side effects in both mother and fetus. For example, β2-agonist (such as Terbutaline and Ritodrine) and calcium blocker (Nifedipine) have been used for tocolytics, but they may complicate the cardiac conditions in fetus receiving the IUBAV.

Of note, the current study has some limitations and several questions still need to be answered. First, to avoid the additional stimulation of control fetus, we did not collect blood samples from the un-operated control fetuses. As a result, we could not compare all of the measurement results between operated and control fetuses at the time of IUBAV completion. In addition, because our primary goal for this study was to control the preterm with all possible approaches, we used combinatorial treatment of IDM and DXS. Thus far, we cannot differentiate the specific effects of IDM from that of DXS on the overall beneficial outcomes in this model. Whether one of the two agents is sufficient to achieve the effects observed in the current study is still unclear. Also, as we discussed above, application of additional tocolytics is necessary to completely control preterm deliver following IUBAV. Lastly, the protocol used in this goat model is similar to the one used for human, but the species difference and the twin-gestational models should be taken into consideration when the results are applied to predict human typical singleton-gestation situation.

## Conclusion

In summary, the administration of IDM/DXS significantly reduces stress response during and after the operation of IUBAV in experimental goats, and this therapy may be valuable in controlling the preterm birth following this intervention in human patients.

## Abbreviation

IUCI, Intrauterine cardiac intervention; S/D, Ratio of peak blood flow between systolic and diastolic phase; LVSP, Left ventricular systolic pressure; IUBAV, Intrauterine balloon aortic valvuloplasty; BAV, Balloon aortic valvuloplasty; BPD, Biparietal diameter; FL, Femur length; HR, Heart rate; LV, Left ventricle; IDM, Indomethacin; DXS, Dexamethasone; NE, Norepinephrine; BW, Body weight; COX, Cyclooxygenase; PG, Prostaglandin.

## Competing interests

The authors declare that they have no competing interests.

## Authors’ contributions

KZ and YH participated in research design and other authors collectively contributed to the performance of laboratory measurements. GW, YL, LZ, RZ, QZ, XH and DM were involved in data collection and analysis. KZ wrote the manuscript. All authors read and approved the final manuscript.
